# Feasibility on the Use of Radiomics Features of 11[C]-MET PET/CT in Central Nervous System Tumours: Preliminary Results on Potential Grading Discrimination Using a Machine Learning Model

**DOI:** 10.3390/curroncol28060444

**Published:** 2021-12-12

**Authors:** Giorgio Russo, Alessandro Stefano, Pierpaolo Alongi, Albert Comelli, Barbara Catalfamo, Cristina Mantarro, Costanza Longo, Roberto Altieri, Francesco Certo, Sebastiano Cosentino, Maria Gabriella Sabini, Selene Richiusa, Giuseppe Maria Vincenzo Barbagallo, Massimo Ippolito

**Affiliations:** 1 Institute of Molecular Bioimaging and Physiology, National Research Council (CNR), 90015 Cefalù, Italy; giorgio.russo@ibfm.cnr.it (G.R.); alessandro.stefano@ibfm.cnr.it (A.S.); albertco1981@gmail.com (A.C.); selene.richiusa@ibfm.cnr.it (S.R.); 2 Nuclear Medicine Unit, Fondazione Istituto G. Giglio, 90015 Cefalù, Italy; barbaracatalfamo@gmail.com (B.C.); cmantarro@gmail.com (C.M.); costanza.longo@virgilio.it (C.L.); 3 Ri.MED Foundation, 90133 Palermo, Italy; 4 Department of Biomedical and Dental Sciences and of Morpho-Functional Imaging, Nuclear Medicine Unit, University of Messina, 98168 Messina, Italy; 5 Neurosurgical Unit, AOU Policlinico “G. Rodolico-San Marco”, University of Catania, 95123 Catania, Italy; roberto.altieri.87@gmail.com (R.A.); cicciocerto@yahoo.it (F.C.); gbarbagallo@unict.it (G.M.V.B.); 6 Interdisciplinary Research Center on Diagnosis and Management of Brain Tumors, University of Catania, 95123 Catania, Italy; 7 Nuclear Medicine Department, Cannizzaro Hospital, 95123 Catania, Italy; sebastiano.cosentino@aoec.it (S.C.); mgabsabini@gmail.com (M.G.S.); ippolitomas@yahoo.it (M.I.)

**Keywords:** positron emission tomography computed tomography, radiomics, machine learning, nuclear medicine, brain tumours

## Abstract

Background/Aim: Nowadays, Machine Learning (ML) algorithms have demonstrated remarkable progress in image-recognition tasks and could be useful for the new concept of precision medicine in order to help physicians in the choice of therapeutic strategies for brain tumours. Previous data suggest that, in the central nervous system (CNS) tumours, amino acid PET may more accurately demarcate the active disease than paramagnetic enhanced MRI, which is currently the standard method of evaluation in brain tumours and helps in the assessment of disease grading, as a fundamental basis for proper clinical patient management. The aim of this study is to evaluate the feasibility of ML on 11[C]-MET PET/CT scan images and to propose a radiomics workflow using a machine-learning method to create a predictive model capable of discriminating between low-grade and high-grade CNS tumours. Materials and Methods: In this retrospective study, fifty-six patients affected by a primary brain tumour who underwent 11[C]-MET PET/CT were selected from January 2016 to December 2019. Pathological examination was available in all patients to confirm the diagnosis and grading of disease. PET/CT acquisition was performed after 10 min from the administration of 11C-Methionine (401–610 MBq) for a time acquisition of 15 min. 11[C]-MET PET/CT images were acquired using two scanners (24 patients on a Siemens scan and 32 patients on a GE scan). Then, LIFEx software was used to delineate brain tumours using two different semi-automatic and user-independent segmentation approaches and to extract 44 radiomics features for each segmentation. A novel mixed descriptive-inferential sequential approach was used to identify a subset of relevant features that correlate with the grading of disease confirmed by pathological examination and clinical outcome. Finally, a machine learning model based on discriminant analysis was used in the evaluation of grading prediction (low grade CNS vs. high-grade CNS) of 11[C]-MET PET/CT. Results: The proposed machine learning model based on (i) two semi-automatic and user-independent segmentation processes, (ii) an innovative feature selection and reduction process, and (iii) the discriminant analysis, showed good performance in the prediction of tumour grade when the volumetric segmentation was used for feature extraction. In this case, the proposed model obtained an accuracy of ~85% (AUC ~79%) in the subgroup of patients who underwent Siemens tomography scans, of 80.51% (AUC 65.73%) in patients who underwent GE tomography scans, and of 70.31% (AUC 64.13%) in the whole patients’ dataset (Siemens and GE scans). Conclusions: This preliminary study on the use of an ML model demonstrated to be feasible and able to select radiomics features of 11[C]-MET PET with potential value in prediction of grading of disease. Further studies are needed to improve radiomics algorithms to personalize predictive and prognostic models and potentially support the medical decision process.

## 1. Introduction

Primary tumours of the central nervous system (CNS) have an incidence of five cases per 100,000 inhabitants/year in Europe and cause 2% of all deaths from cancer. In recent decades, there has been a progressive increase in incidence, not only for the larger diffusion of improved imaging methods that allow a more accurate diagnosis, but a significant increase was noted in the age group >65 years, where the incidence has more than doubled [1]. Gliomas represent more than 80% of CNS primary tumours in adults and the largest part of them are Glioblastoma (GBM) [2]. The classification of CNS tumours was significantly reorganized in the 2016 and 2021 WHO update [3,4]. 

Accordingly, it is now recognized that certain molecular and genetic features of tumours, in some instances, can be more important in response prediction and prognosis than histological type and grade, which has obvious implications in clinical therapy and research [5].

CNS tumour grading has, for many decades, differed from the grading of other, non-CNS neoplasms, since brain and spinal cord tumours have had grades applied across different entities. For the latest version of the WHO classification of CNS tumours, grade of disease was correlated to an idealized clinical-biological behaviour; for instance, WHO grade I tumours were curable if they could be surgically removed; at the other end of the spectrum, WHO grade IV tumours were highly malignant, leading to death in relatively short periods of time in the absence of effective therapy [4]. In view of this classification, the importance of having a tool able to predict the grade of disease at diagnosis of CNS tumours has become a crucial point of clinical interest. 

Historically, in radiology practice, trained physicians visually assessed medical images for the detection, characterization, and monitoring of diseases. In the last decade, the use of radiomics in the study of medical images has aroused increasing interest. The workflow of radiomics is fundamentally based on image acquisition and reconstruction, tissue segmentation, feature extraction and selection, and finally analysis of the data obtained [6]. The fundamental hypothesis of radiomics is that quantitative analysis of tumours through the large amount of features can provide valuable diagnostic, prognostic or predictive information. Numerous studies have demonstrated the correlation between the heterogeneity of the tissues and the radiomics features, which would allow obtaining relevant information through the analysis of the images alone [7,8]. 

Over the years, the clinical role of positron emission tomography (PET) has evolved considerably, especially in the evaluation of cancer patients in the field of nuclear medicine. Although the ability to discriminate between high-grade and low-grade CNS tumors may be evaluated with [18F]-Fluorodeoxiglucose (FDG) PET, in our research we investigate the diagnostic power of brain PET images acquired through [11C]-methionine (MET), which shows potential advantages, through a radiomics approach. In this field, Alongi et al. [9] shown that several radiopharmaceutical agents including MET present potential utility in the differential diagnosis, estimation of prognosis, and evaluation of recurrence and treatment planning in patients with glioma. The value of MET-PET was demonstrated in a meta-analysis performed by A. H. Katsanos et al. defining the best performances for this diagnostic method in the evaluation of grading in gliomas [10]. The authors included many study protocols (8 used FDG PET, 4 FET PET, and 6 MET PET, 3 both FDG PET and FET PET), demonstrating the higher sensitivity of MET-PET, followed by FET PET for the detection of glioma grade [11,12,13,14,15,16,17,18,19,20,21,22,23,24,25,26,27,28,29,30,31,32,33]. MET-PET has the best available evidence with 95% of sensitivity and 95% of specificity. 

Nowadays, the literature is missing radiomics studies that include end points based on radiomics evaluation of prediction of disease grading in CNS tumours using MET-PET. In this regard, the aim of the present study is to evaluate the feasibility of ML on 11[C]-MET PET/CT scan images and to propose a radiomics workflow, using a machine-learning method to create a predictive model capable of discriminating between low-grade and high-grade CNS tumours.

## 2. Materials and Methods

### 2.1. Patient Dataset and Protocol Acquisition 

Being a retrospective study, the first part of the work was the collection of images. The selection criteria involved patients who underwent MET-PET examinations at completion of conventional imaging for diagnosis/staging and whose subsequent histopathological results were known. Histological examinations identified “low grade” (0) or “high grade” (1) CNS tumours based on the 2016 WHO Classification. Fifty-six patients were selected from January 2016 to December 2019. The temporal range has been defined to have homogenous protocol data. The image acquisition protocol was as follows: the examination was performed 10 min after the administration of 11C-Methionine; the exam lasted 15 min; the activity administered was recorded in the range of (401–610) MBq. It was decided to work with two groups of images acquired by two different scanners to increase the statistics: 24 patients by the Biograph Horizons scanner (Siemens Healthineers, Erlangen, Germany) and 32 patients by the Discovery 690 scanner (General Electric Medical Systems). Images for the Biograph Horizons scanner were reconstructed with a 512 × 512 image matrix and a voxel size of 0.4821 mm × 0.4821 mm × 3 mm using manufacturer point spread function correction and time-of-flight (TOF) using Ordered Subset Expectation Maximization (OSEM) with 4 iterations. For the Discovery 690 scanner images were reconstructed with a 256 × 256 image matrix, with a grid spacing of 1.17 mm and a thickness of 3.27 mm using OSEM with the two-iterative process. 

The histological characteristics and tumour grading of the selected patients are summarized in the following table (Table 1). Specifically, in the whole patients’ dataset, 39 high-grade e 17 low-grade tumours were considered. Considering the subgroup of 24 patients who underwent Siemens tomography scans, tumours were differentiated in 16 high-grade (13 glioblastoma, 3 anaplastic astrocytoma), and 8 low-grade (3 pilocytic astrocytoma, 4 oligodendroglioma, and 1 ganglioglioma). In the subgroup of 32 patients who underwent GE tomography scans, tumours were differentiated in 23 high-grade (20 glioblastoma, 3 anaplastic astrocytoma), and 9 low-grade (5 diffuse astrocytoma, 2 oligodendroglioma, and 2 meningioma). The ratio of high-grade to low-grade tumours was slightly unbalanced all groups: high-grade were about 70% of the tumours considered for each group of patients, reflecting the current clinical scenario for whom PET imaging is usually requested.

Patient images and information have been anonymized in accordance with the Italian privacy law.

### 2.2. Overview of the Proposed Radiomics Workflow

**Figure 1:**
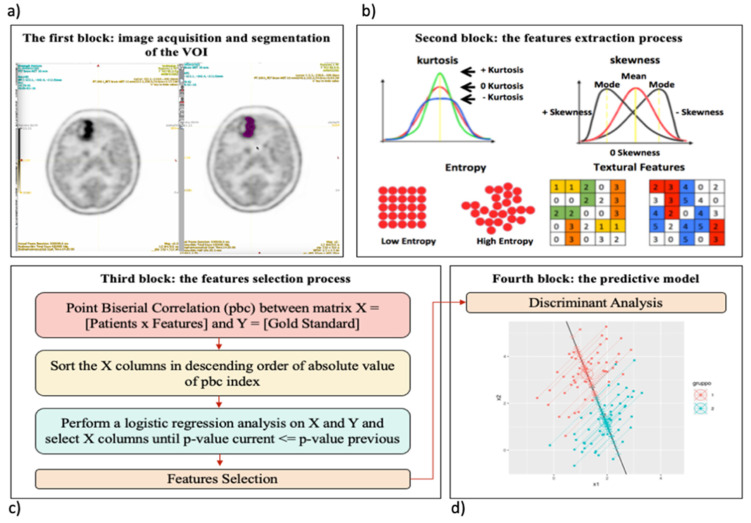
The radiomics workflow of the study. (**a**) PET image with lesion segmentation based on the thresholding method (see Section 2.1). (**b**) Some examples of the 44 features extracted using LifeX software, such as kurtosis, skewness, entropy and two textural features in the form of matrix (see Section 2.2), (**c**) Feature selection using a mixed descriptive-inferential sequential approach (see Section 2.2.3), (**d**) Representative figure of biplanar feature classification (low and high tumour grade) using the discriminant analysis as the supervised classifier. Specifically, a set of labelled instances (low and high tumour grade) were used for training purpose. Successively, the classifier was used to predict the label in the test set, where instances were without corresponding labels (see Section 2.2.4).

We implemented a radiomics workflow (Figure 1) to identify a relevant prognostic model in patients with brain tumors who underwent 11[C]-MET PET/CT examinations. 

Briefly, (a) two different segmentation algorithms were applied for each PET study to identify the lesion in a user-independent way, as explained in Section 2.1, to avoid intra-observer and inter-observer dependencies concerning manual delineation; (b) the Local Image Feature Extraction (LifeX) software [34] was used to extract 44 radiomics features for each lesion, as explained in Section 2.2; (c) a mixed descriptive-inferential sequential approach [6] was used to identify a subset of relevant features that correlate with the outcome, as explained in Section 2.2.3; (d) a machine learning model was built to discriminate between tumor grades. A variety of different classifiers may be used for this last step (neural networks, random forest, support vector machines, and generalized linear models); in this study, the supervised discriminant analysis classification algorithm was used, as explained in Section 2.2.4. In the following, a detailed discussion of the various steps is presented.

#### 2.2.1. The Target Segmentation 

**Figure 2:**
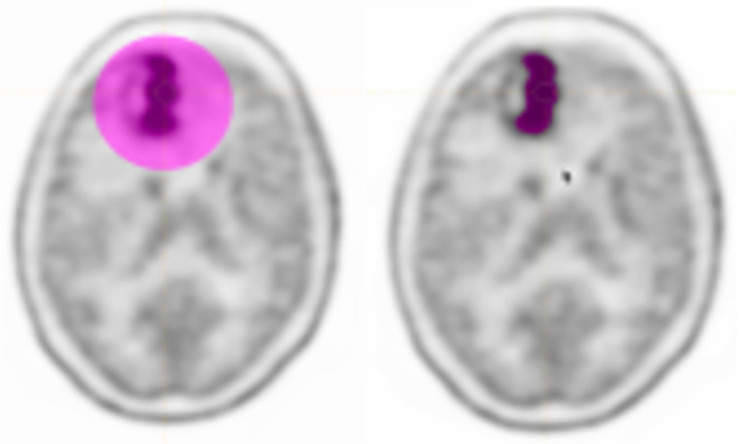
Initial user input and result of segmentation (Example patient).

The LIFEx software was used to perform the lesion delineation step in the radiomics workflow. Image pre-processing was adopted to reduce noise, improve image characteristics such as edges, and sample homogenization. As suggested in [35], to obtain more robust results, we resampled using 64 bins between 0 and 20 Standardized Uptake Value (SUV) units (i.e., size bin equal to 0.317) being this one the most frequent SUV range in oncology. For spatial resampling, voxel size remained that of the original images. At this point, the nuclear medicine physicians identified the tumour region and proceed with the delineation. Although manual delineation seems the most intuitive and easily implemented way of obtaining lesion area, it is time-consuming and subject to inter- and intra-observer variability [36]. For this reason, the thresholding segmentation method was used. In the images acquired with 11C-methionine, major structures (brain tumours) were well-distinguished with respect to surrounding tissues and the threshold method was sufficient to identify the lesion area. The area containing the target was localized by the operator and a SUVmax threshold value of 40% [37] was used to identify the volume of interest (VOI), see Figure 2. The user input was minimal and limited at drawing a rough contour around the target to avoid false positives.

**Figure 3:**
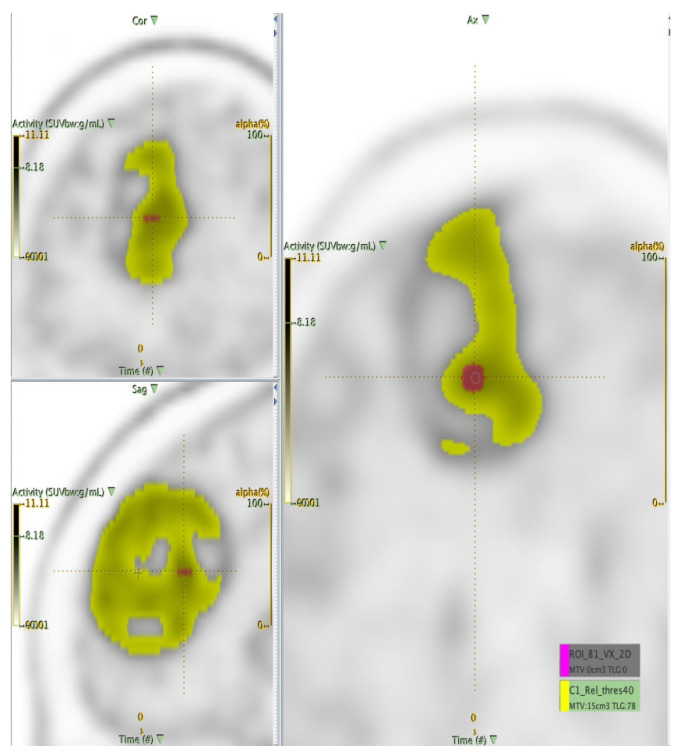
Illustration of volume segmentation (fixed ROI in purple vs. VOI in yellow).

In addition, to eliminate the variability of the volume, for each patient study another segmentation of fixed size was considered: a region of interest (ROI) of 81 voxels centred on the SUVmax voxel was identified. Finally, radiomics features were extracted from the ROIs and VOIs (Figure 3), as described in the following section.

#### 2.2.2. Extraction of the Radiomics Features

LIFEx software was used to calculate 44 radiomics features starting from the VOIs and ROIs to quantify the characteristics of the tumour. Features reflecting the shape, the voxel values, the histogram, and the textural content are divided into first, second, and third order to provide information related to the grey-level distribution without considering spatial relations between voxels (first order), considering the spatial relations of voxels (second order), and evaluating spatial relationship among three or more voxels (third order). Specifically, a set of 31 texture indices (7 from grey-level co-occurrence matrix GLCM, 3 from the neighbourhood grey-level different matrix NGLDM, 10 gray level run length matrix GLRLM, 11 grey-level size zone matrix GLZLM), 4 histogram indices—HISTO Skewness, HISTO Kurtosis, HISTO Energy, HISTO Entropy log10-, four shape indices—SHAPE Sphericity, SHAPE Compacity, SHAPE Volume mL and voxels—and 5 conventional features—CONVmin, CONVmean, CONVmax, CONV TLG, CONV RIM—were computed from each VOI and ROI. A detailed description of radiomics features is available in the LIFEx manual [34]. The extracted features were then inserted into a matrix consisting of 2*n rows (where *n* = 56, the patients’ number) and m columns (where m = 44, the feature number), each associated with the relative gold standard, 0 and 1 indicating low grade or high-grade tumours, respectively.

#### 2.2.3. Statistical Analysis: The Feature Selection Process

Not all extracted features contain information relevant to the identification of a relevant prognostic model. For this reason, through statistical analyses, it is possible to identify a subset of relevant features related to the goal, in order to eliminate the redundancy and reduce the dimensionality of the problem to improve the accuracy of the prediction. This task is obtained using a mixed descriptive-inferential sequential approach proposed by Comelli et al. [35] based on the biserial correlation point (pbc), which calculates the correlation between the single feature and the dichotomous variable, i.e., the gold standard 0 and 1. Consequently, the features are sorted from highest to lowest pbc and a while loop is implemented to perform a logistic regression for each feature. Considering the *p*-value as the output of the logistic, the smaller the *p* value, the better the logistic regression model that interprets the gold standard. The process is killed when the *p*-value does not decrease. Finally, the features with valuable association with tumour grading were identified. A more detailed description is reported in [35]. 

#### 2.2.4. The Predictive Model

The last step is to create the classification model. A supervised classifier uses a set of labelled instances for training purposes. Labels represent the categories of interest, low and high tumour grade. Successively, the classifier is used to predict the label in the test set, where instances are without corresponding labels. This is equivalent to building an algorithm that analyses training data and provides a hypothesis (i.e., function), which can be used to predict new outputs.

In this study, a supervised classification algorithm based on discriminant analysis [38] was used to overcome the data imbalance issue described in Section 2.1. Specifically, considering that, in a current clinical scenario, high-grade tumours are more frequent than low-grade ones, our dataset was unbalanced (approximately 70% of high-grade tumours and 30% of low-grade tumours). Consequently, discriminant analysis was chosen because unbalanced datasets do not have a negative effect on the performance of the discriminant analysis [39]. Furthermore, if only a limited amount of data is available, such as in our study where 56, 24, and 32 tumours were found in the three patient subgroups described in Section 2.1, the k-fold cross-validation strategy can be used. In this way, each dataset was divided into k subsets and one of the k subsets was used as a validation set and the other k-1 subsets were brought together to form the training set. This process was repeated k times. The grouping was done so that both the training and validation sets maintained the same lesion grade percentage as the original dataset to maintain the proportion present in a real clinical environment where high-grade tumours are more frequent than low-grade ones. Then, the average error across all experiments is computed. In this way, (i) overfitting and asymmetrical sampling are avoided increasing the accuracy of final results, (ii) several models can be tested, and (iii) the results averaged over all the folds are more robust. 

Finally, the diagnostic performance of each prediction model was calculated, including specificity, sensitivity, accuracy and area under the receiver operating characteristic curve (AUC). In particular, specificity was calculated to indicate the ability to correctly identify subjects with high-grade, sensitivity to indicate the ability to correctly identify subjects with low-grade, accuracy to provides the percentage of total elements classified successfully, AUC to summarize the overall diagnostic accuracy of the test. AUC takes values from 0 to 1, where a value of 0 indicates a perfectly inaccurate test and a value of 1 reflects a perfectly accurate test. In general, an AUC of 0.5 suggests no diagnostic discrimination, 0.7 to 0.8 is considered acceptable, 0.8 to 0.9 is considered excellent, and more than 0.9 is considered outstanding.

## 3. Results

Starting from the 44 features obtained for each ROI and VOI, the reduction and selection method described in Section 2.2.3 was capable to identify the most relevant features on the two segmentations, i.e., the VOI obtained using the thresholding method and the fixed ROI (see Section 2.1). Table 2 and Table 3 show the features more significantly correlated with the histopathological results in both segmentation cases for the three patient groups (the whole patients’ dataset, patients who underwent GE scan, and patients who underwent Siemens scan). Although some selected features show a *p*-value greater than 0.05, we considered it more appropriate to proceed with the selection of the features and with the implementation of the relative predictive model to have a complete representation of the results, while remaining aware of this statistical anomaly. 

Finally, Table 4 and Table 5 show the performance evaluation in the predictive model obtained using the discriminant analysis (Section 2.2.3). Specifically, considering the fixed ROI, our model was able to predict tumour grade with the best accuracy (~73%, AUC ~ 79%) in the subgroup of patients who underwent Siemens tomography scans, while the worst condition occurred in the whole patients’ dataset (accuracy = 57.25%, AUC = 58.51%). In the same way, considering the VOI, the best model was obtained in the Siemens sub dataset (accuracy ~85%, AUC = ~79%), while the worst was obtained in the whole patients’ dataset (accuracy = 70.31%, AUC = 64.13%).

**Figure 4:**
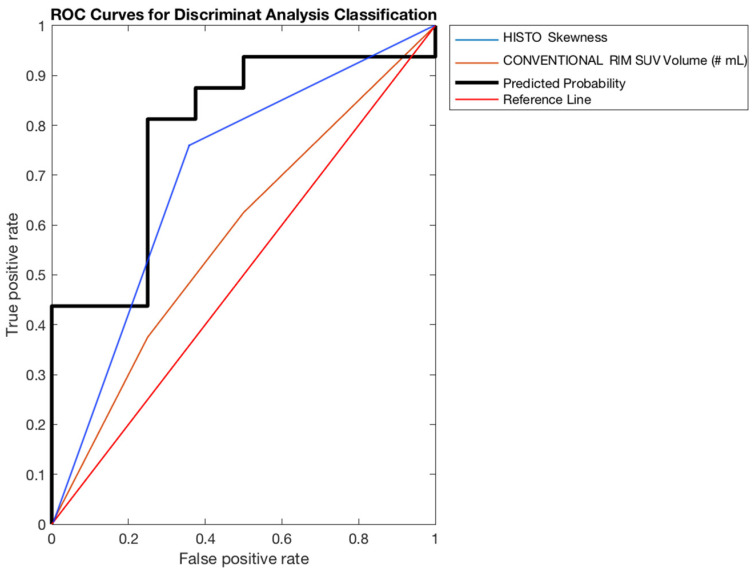
ROC curve for fixed ROI (SIEMENS patients’ group).

**Figure 5:**
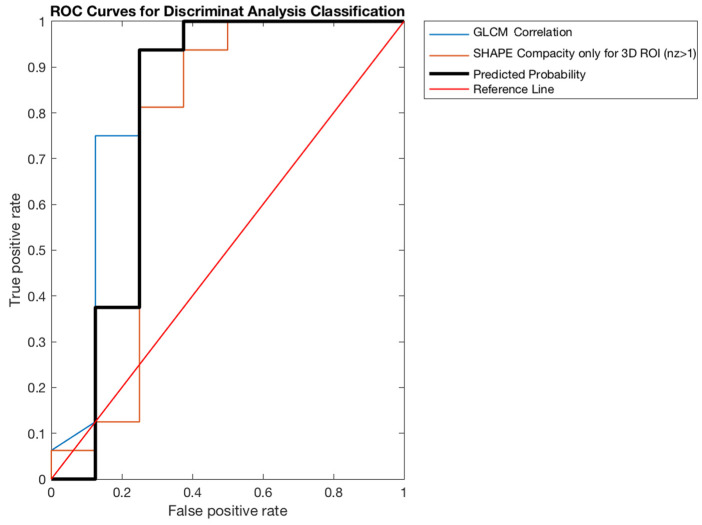
ROC curve for VOI (SIEMENS patients’ group).

In both cases, the group showing the highest values, therefore the best, was the one related to Siemens tomograph; its areas under the curves (AUCs) are shown in Figure 4 and Figure 5. 

## 4. Discussion

In the present study, an efficient and repeatable radiomics approach in the evaluation of CNS tumours grading was evaluated combining a panel of features that were potentially able to differentiate between low-grade and high-grade CNS tumours.

According to the latest guidelines for imaging glioma [39], one of the main indications for using PET with radiolabelled amino acids and 2-[18F]fluoro-2-deoxy-d-glucose ([18F]FDG) at the time of diagnosis is the differentiation of high-grade glioma (HGG) from low-grade glioma (LGG) or nonneoplastic lesions. On 18F-FDG-PET imaging, Grade I/II gliomas typically have FDG uptake similar to or less than white matter uptake, although some grade I/II gliomas such as pilocytic astrocytomas have high FDG uptake. Grade III/IV gliomas typically have FDG uptake greater than white matter uptake. For 18F-FET-PET imaging an early peak in the TAC shape of the mean ROI/VOI activity (<20 min after injection) followed by a plateau or a decreasing TAC is indicative of a grade III/IV tumour. However, available data on the range of radiotracer uptake are limited to FDG, FET and MET. For all of these radiopharmaceuticals agents when uptake level remains in the background uptake range or slightly above excludes a grade III/IV glioma, lymphoma or metastasis with high probability. Additionally, an oligodendroglial tumour is very unlikely. A grade I/II astrocytoma cannot be excluded since approximately 30% exhibit low uptake. Conversely, an increased uptake has high a positive predictive value for a neoplastic process. Nevertheless, a reliable differentiation of grade III/IV and grade I/II gliomas is not completely possible because of a high proportion of active tumours among the latter, especially in oligodendrogliomas. Local areas with the highest uptake should be used for biopsy guidance [40].

The first goal of the study was to demonstrate the feasibility of the use of an innovative statistical methodology for the selection of significant descriptors proposed in a previous article [35], using a solid statistical classifier, e.g., the discriminant analysis, to create a predictive model potentially capable to discriminate between low-grade and high-grade CNS tumours using MET-PET studies. Specifically, we used the discriminant analysis [41] as machine learning algorithm because unbalanced datasets do not have a negative effect on the discriminant analysis performance [39]. In our study, high-grade tumours are more frequent than low-grade ones reflecting the current clinical scenario for whom PET imaging is usually requested, so we keep the same percentage during the k-fold strategy. In addition, the sensitivity and specificity values found for Siemens patients (Table 4 and Table 5), seem to confirm the goodness of the results obtained despite the unbalanced database. The present model consists of the use of two different segmentation algorithms that were used to identify the lesion avoiding intra- and inter-user variability that may occur with manual delineation: (i) a VOI obtained using an automatic thresholding method, and (ii) a fixed ROI of 81 voxels centred on the SUVmax voxel to eliminate the dependence on the volume. Since the highest performance was obtained using the first method (i.e., VOI), we can conclude that there are no particular advantages in using a homogeneous region of interest (e.g., fixed ROI) rather than a segmentation algorithm that obtains different volumes among patients’ studies. In particular, the results showed that our workflow achieves good performance, in terms of accuracy (greater than 70.31%) and specificity (greater than 71.81%), in all cases except when we considered both scanners using the fixed ROI.

However, the performance values for AUC were sub-optimal (<78.91%) most probably due to the limited number of patients and unbalancing of low vs. high-grade tumours evaluated in different tomographs. For better performance, a machine learning algorithm should work with the same number of “objects” for both classes (low-grade and high-grade tumours). In our work, the group that showed the most symmetry was the one related to patients acquired on Siemens scanner, and, consequently, we noticed higher performance (accuracy ~85%). In addition, radiomics features are highly related to scanner properties, acquisition parameters, reconstruction algorithm, slice thickness, voxel size, and image processing thus hampering multicentre studies. Consequently, different features have been extracted considering the two different scanner subgroups (Siemens and GE tomographs). Standardization, in this context, involves implementing a defined procedure for the radiomics process, including a post-extraction correction, that is applied directly to the derived radiomics features, as opposed to the pre-extraction image values [42]. Consequently, a decrease in performance was to be expected when the combination of the subgroups was evaluated in this retrospective analysis. Specifically, the features selected are probably less affected by significant variability among the two scanners. Further studies assessing harmonization strategies are needed to improve our results. However, the results presented support the reproducibility of radiomics extraction on MET-PET images, independently by operator and based on features selected, probably also by PET scanners. These results should become relevant for the correct planning of further studies. In fact, despite the great success of radiomics, several issues are not properly addressed in various published studies. In particular, (i) operator-independent segmentation methods must be used to extract target volumes in order to avoid irreproducible results, (ii) ad hoc high-throughput analysis tool IBSI (Image Biomarker Standardization Initiative) compliant must be used, such as LifeX, to extract features automatically, (iii) feature selection methods must be implemented in order to eliminate the redundancy and reduce the dimensionality of the problem, (iv) a classification method supported by solid statistical reasoning must be implemented to avoid empirical strategies. All these issues were opportunely addressed in our study.

Other studies with different approaches, defined the potential use of radiomics on FET-PET imaging to differentiate MS from glioma II°–IV° or on 18F-DOPA-PET to predict pathologic Methylation of the O6-methylguanine methyltransferase (MGMT) gene promoter status in gliomas [43,44]. Future radiomics trials should enhance the results in the present preliminary study but a real goal would regard in the future the association of MET-PET and MRI images data as demonstrated by Kebir et al. using a semiquantitative approach (T/B ratio) for prediction of isocitrate dehydrogenase (IDH) mutation in classifying glioma [45].

This preliminary study, for the brief results obtained, presents different limitations. Despite our selection of different diseases (Gliomas, Astrocytomas, Meningiomas) that may reflect the clinical scenario of diseases that can occur daily using MET-PET, the first results reported might be affected by the inhomogeneous and relatively small population. Further study with more analysis such as comparative results of machine learning model and MRI images, or with visual inspection or semiquantitative TBR analysis, in a larger cohort of patients (more than one hundred patients and with sub-groups analysis) will be performed using the same model, also by correlating the radiomics and clinical/genetics features for all most frequent CNS tumours.

## 5. Conclusions

This preliminary study on radiomics featuring an analysis using a machine learning model suggests the feasibility of the application of ML on 11[C]-MET PET images and a potential prediction of grading discrimination at diagnosis. Further studies are needed to improve radiomics algorithms to personalize predictive and prognostic models and potentially support the medical decision process.

## Figures and Tables

**Table 1:** Characteristics of population.

Histological Diagnosis	WHO Grade	Number
Glioblastoma	IV	33
Anaplastic astrocytoma	III	6
Diffuse astrocytoma	II	5
Oligodendroglioma	II	6
Pilocytic astrocytoma	I	3
Ganglioglioma	I	1
Meningioma	I	2

**Table 2:** Relevant features using the fixed ROI. NGLDM: neighbourhood grey-level different matrix; GLZLM LZLGE: grey-level zone length matrix long-zone low grey-level emphasis; GLRLM LRLGE: gray level run length matrix long run low grey-level emphasis; CONV RIM: Conventional ROI Intensity Mean.

Tomograph	Features	*p*-Value
All patients	NGLDM Busyness	0.1615
	GLZLM LZLGE	0.3207
GE	GLRLM LRLGE	0.05
	GLZLM LZLGE	0.137
SIEMENS	Histogram Skewness	0.0136
	CONV RIM SUV Volume	0.0136

**Table 3:** Relevant features using the VOI. GLRLM LRLGE: Gray Level Run Length Matrix Long Run Low Gray-Level Emphasis; LGRE: Low Gray-level Run Emphasis; GLCM: Grey Level Co-occurrence Matrix.

Tomograph	Features	*p*-Value
All patients	Shape Sphericity	0.0314
	Shape Compacity	0.0215
	Histogram Kurtosis	0.0232
GE	GLRLM LRLGE	0.0481
	GLRLM LGRE	0.117
SIEMENS	GLCM Correlation	0.00036
	Shape Compacity	0.0014

**Table 4:** Performance values of the predictive model for Fixed ROI.

Tomograph	Sensitivity	Specificity	Accuracy	AUC (95%CI)
All patients	41.17%	63.60%	57.25%	58.51% (41.75–75.27%) 62.80% (39.15–86.45%) 78.91% (58.80–99.01%)
GE	28.52%	88.47%	71.64%	
SIEMENS	76.67%	71.81%	72.88%	

**Table 5:** Performance values of the predictive model for VOI.

Tomograph	Sensitivity	Specificity	Accuracy	AUC (95%CI)
All patients	52.44%	76.62%	70.31%	64.13% (49.76–80.50%) 65.73% (40.04–89.43%) 78.91% (51.96–105.85%)
GE	71.76%	83.76%	80.51%	
SIEMENS	86.67%	84.86%	84.98%	

## Data Availability

The data presented in this study are available on request from the corresponding author. The data are not publicly available.
